# Supplementary material for: Human immunoglobulin G hinge regulates agonistic anti-CD40 immunostimulatory and antitumour activities through biophysical flexibility
Source: Nat Commun. 2019 Sep 27;10:4206. doi: 10.1038/s41467-019-12097-6 (PMC6765011; doi:10.1038/s41467-019-12097-6)
Supplement: Supplementary file 1 — Supplementary Information [file 41467_2019_12097_MOESM1_ESM.pdf]

Supplementary Information

Human immunoglobulin G hinge regulates agonistic anti-CD40 immunostimulatory and antitumour activities through biophysical flexibility

Liu et al.

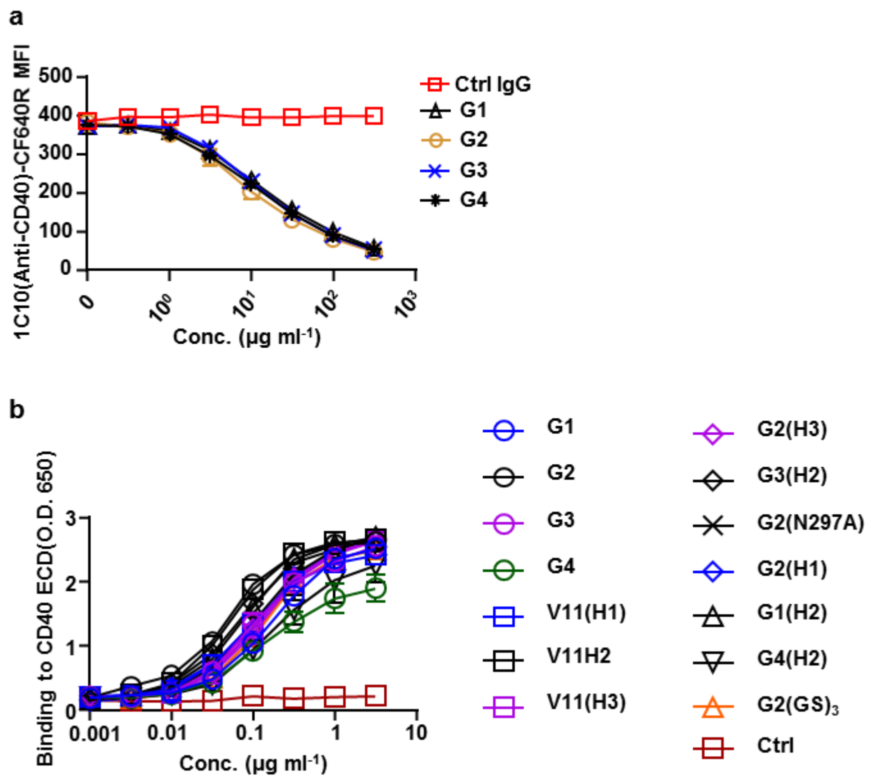

Supplementary Fig. 1. Binding of various anti-mouse CD40 antibodies to mouse CD40. (**a**) Levels of 1C10 (anti-mouse CD40)-CF640R staining (MFI) in FcγR$^{-/-}$ B cells in the presence of control or 1C10-derived antibodies of indicated constant domains. Splenocytes of FcγRα$^{-/-}$ mice were stained with CF640R-conjugated 1C10 in the presence of PE-conjugated anti-B220 and various amounts of control IgG or anti-CD40 antibodies of indicated constant domains. Mean fluorescence intensity (MFI) of 1C10(anti-CD40)-CF640R staining in B220^{+} cells were plotted against the concentration of control IgG or the competitive anti-CD40 antibodies of indicated constant domains. (**b**) Binding of anti-CD40 antibodies to mouse CD40 extracellular domain (ECD) quantified as ELISA O.D. values. Binding of control and anti-CD40 antibodies was detected by HRP-conjugated anti-human IgG. Absorbance at 650nm was recorded and plotted against the concentration of control IgG or the anti-CD40 antibodies of indicated constant domains. Bars represent the mean ± SEM. Source data are provided as a Source Data file. A representative of two independent experiments is shown.

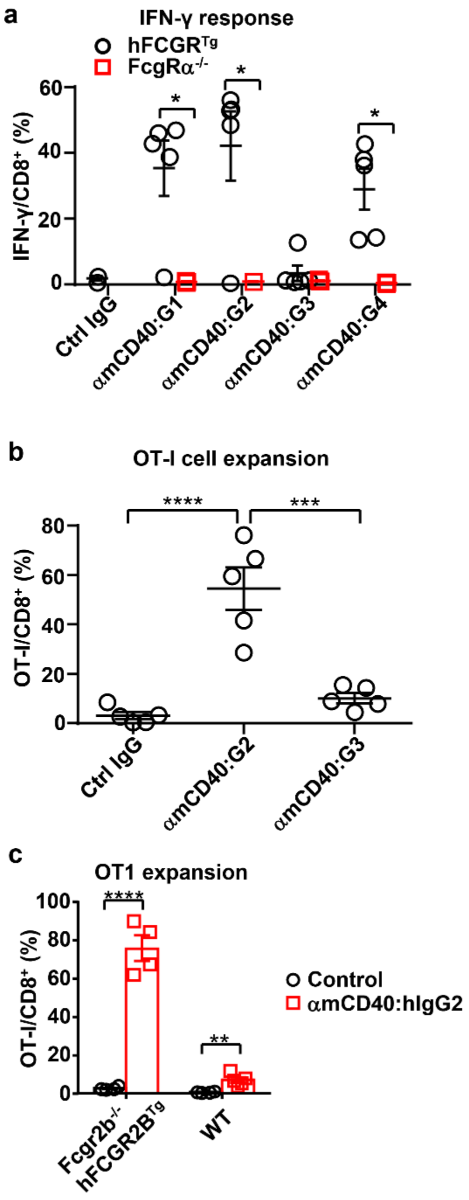

Supplementary Fig. 2. Agonistically inactive property of IgG3 and suboptimal IgG2 agonism in WT mice. (**a**) Quantification of IFN-γ$^+$ cells among CD8$^+$ T cells upon OVA peptide SIINFEKL stimulation in FcγR-humanized and FcgRα$^{-/-}$ mice treated and analyzed as in Fig. 1b together with indicated control or anti-mCD40 antibodies of indicated constant domains (30 µg per mouse). Numbers of mice: 3 hFCGR^{Tg} mice for Ctrl IgG, 5 hFCGR^{Tg} and 3 FcγRα$^{-/-}$ mice for other groups. (**b**) Quantification of OT-I cells in FcγR-humanized mice treated and analyzed as in Fig. 1a together with 100 µg of control IgG, 10 µg of IgG2 anti-CD40 antibody, or 100 µg of IgG3 anti-CD40 antibody. Numbers of mice: 5 mice per group. (**c**) Quantification of OT-I cells in FcγRIIB-humanized and WT mice as treated and analyzed in Fig. 1a together with indicated antibodies (10 µg per mouse). Numbers of mice: 4~5 mice per group. Each symbol represents an individual mouse. Bars represent the mean ± SEM. * $p \leqslant 0.05$, ** $p \leqslant 0.01$, *** $p \leqslant 0.001$, **** $p \leqslant 0.0001$, unpaired two-tailed t-test (**a**, **c**), and one-way ANOVA with Holm-Sidak's post hoc (**b**). Source data are provided as a Source Data file. A representative of two independent experiments is shown (**a**, **c**).

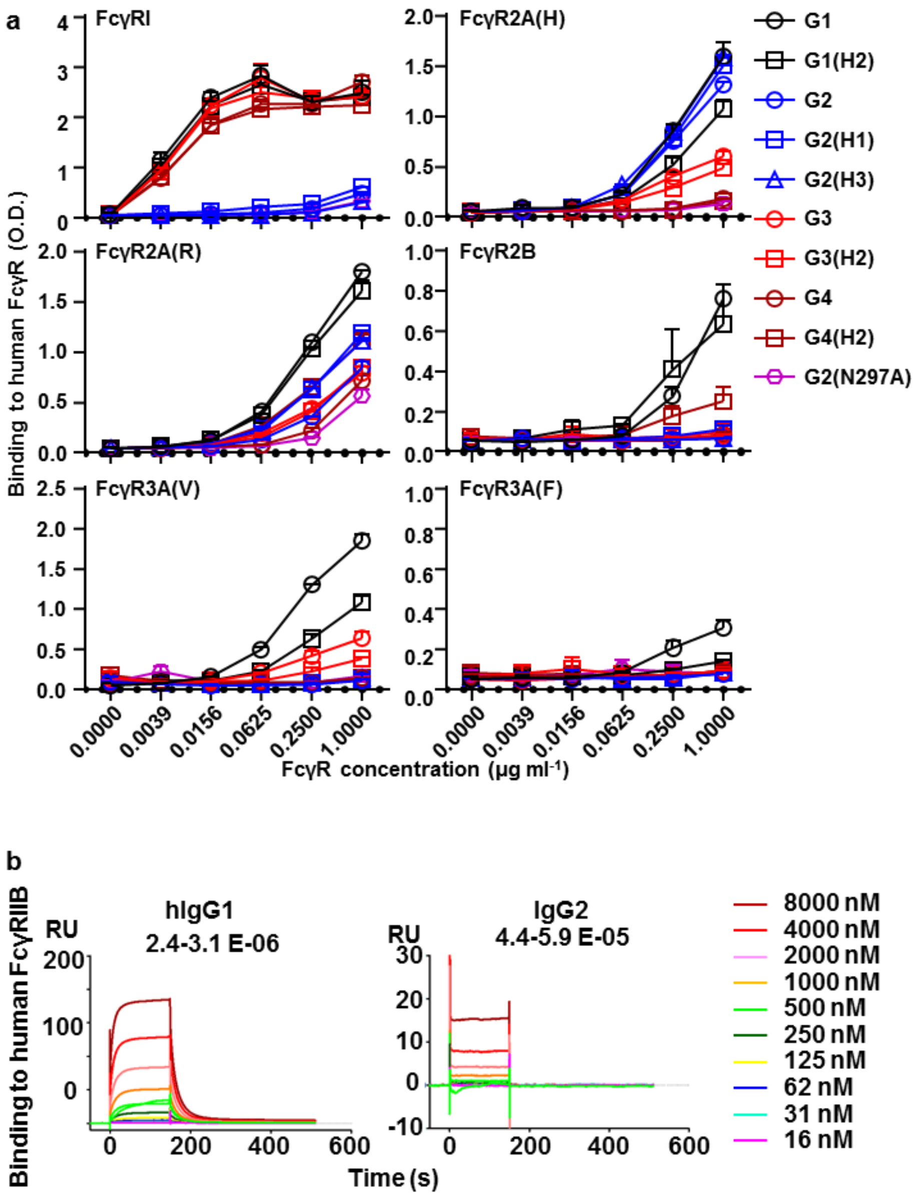
a
FcγRI
FcγR2A(H)
FcγR2A(R)
FcγR2B
FcγR3A(V)
FcγR3A(F)
Binding to human FcγR (O.D.)
FcγR concentration (μg ml-1)
0.0000
0.0039
0.0156
0.0625
0.2500
1.0000
G1
G1(H2)
G2
G2(H1)
G2(H3)
G3
G3(H2)
G4
G4(H2)
G2(N297A)
b
Binding to human FcγRIIB
hIgG1
2.4-3.1 E-06
IgG2
4.4-5.9 E-05
RU
Time (s)
8000 nM
4000 nM
2000 nM
1000 nM
500 nM
250 nM
125 nM
62 nM
31 nM
16 nM

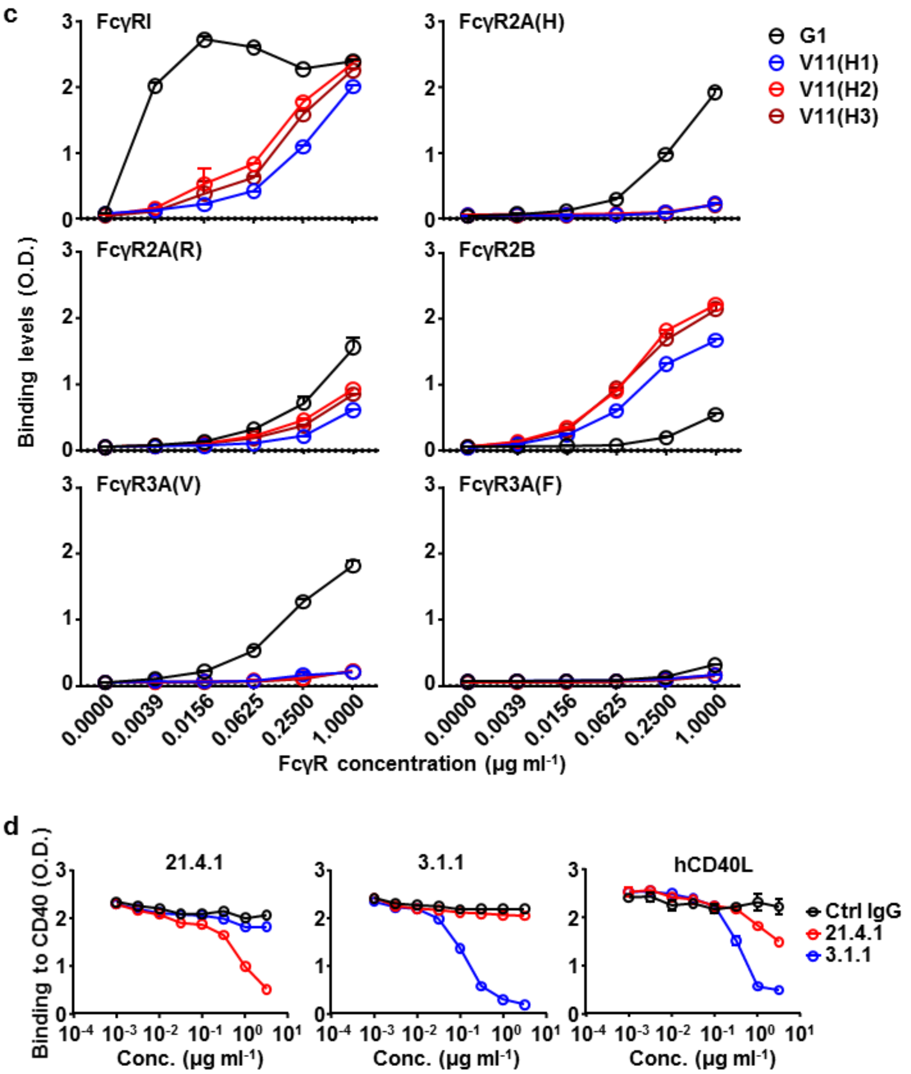

Supplementary Fig. 3. Comparable FcγR binding properties of IgG hinge variants. (**a, c**) Binding of anti-CD40 antibodies of indicated different natural (G1, G2, G3 and G4), chimeric (G1(H2), G2(H1), G2(H3), G3(H2) and G4(H2)), and mutant (G2(N297A), V11(H1), V11(H2) and V11(H3)) constant domains to indicated human FcγRs were evaluated in ELISA. Binding of biotinylated FcγRs to coated anti-CD40 antibodies of indicated constant domains was detected by HRP-conjugated streptavidin. Absorbance at 650nm was recorded and plotted against the concentration. **(b)** SPR analysis of the binding of human IgG1 and 2 antibodies to human FcγR2B. Presented are real-time sensorgrams with affinity constants (KD). (**d)** Clone 21.4.1 and 3.1.1 have

different binding epitopes on human CD40. Presented are binding of 21.4.1, 3.1.1 and human CD40L (hCD40L) to immobilized human CD40 extracellular domain (ECD) preincubated with control (Ctrl IgG), 21.4.1 or 3.1.1 antibodies, quantified by ELISA signals (O.D.). Bars represent the mean ± SEM. Source data (**a, c, d**) are provided as a Source Data file.

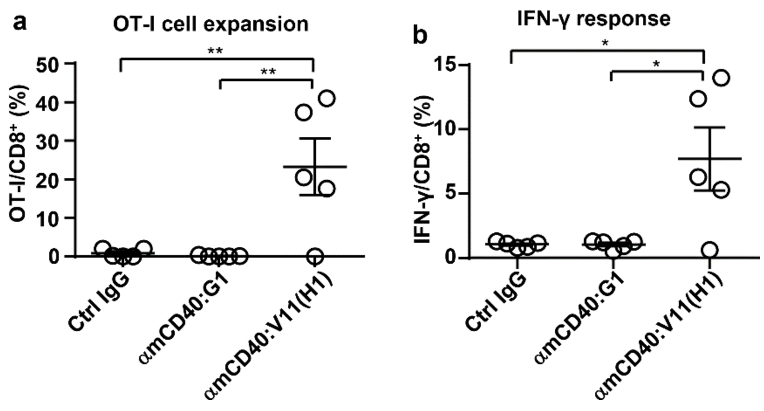

Supplementary Fig. 4. Enhanced agonistic potency of V11 Fc. (**a**-**b**) Quantification of OVA-specific OT-I cells (**a**) and IFN-γ$^+$ cells (**b**) in FcγR-humanized mice (5 mice per group) treated and analyzed as in Fig. 1a and Supplementary Fig. 2a together with control or anti-CD40 antibodies of indicated IgG constant domains (3.16 μg per mouse). Each symbol represents an individual mouse. Bars represent the mean ± SEM. * $p \leqslant 0.05$, ** $p \leqslant 0.01$, one-way ANOVA with Holm-Sidak's post hoc. Source data are provided as a Source Data file. A representative of two independent experiments.

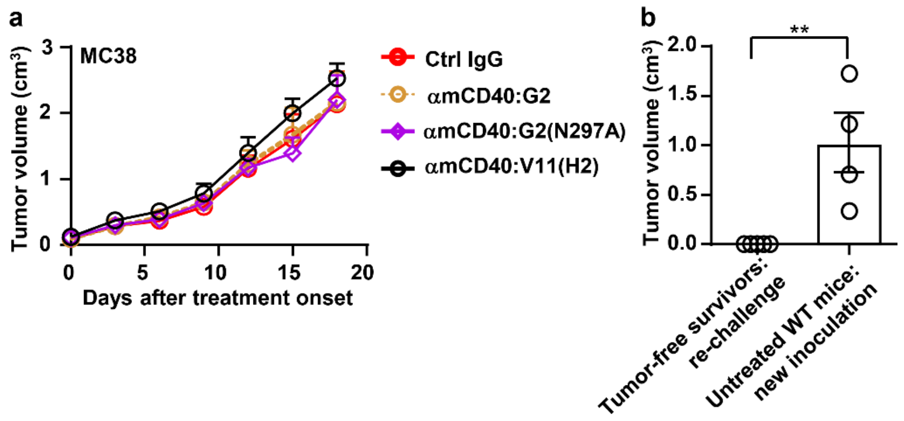

Supplementary Fig. 5. The FcγR-binding requirement for agonistic anti-CD40 antitumor activities. (a) Shown are MC38 tumour growth curves in FcγR-deficient (FcgRα$^{-/-}$) mice following treatment of anti-mouse CD40 antibodies of indicated constant domains. After MC38 tumour cells were subcutaneously inoculated and established (set to be day 0), mice (6 per group) were treated *i.p* twice on day 0 and 3 with 31.6 μg/mouse of control or anti-CD40 antibodies of indicated constant domains, and monitored for tumour growth. Bars represent the mean ± SEM. **(b)** MC38 tumour volumes in control and tumour-free survivors after tumour re-challenge. Five FcγR-humanized mice that had been treated with V11(H2) and rejected MC38 tumours in Fig. 3a, referred to as "tumour-free survivors", were re-challenged with MC38 tumour cells 60 days after the initial treatment and analyzed for tumour volumes 23 days after re-challenge. At the same time, MC38 cells used for re-challenge were also inoculated in four untreated WT control mice and monitored for tumour growth. Bars represent the mean ± SEM. ** $p \leqslant 0.01$, unpaired two-tailed *t*-test. Source data are provided as a Source Data file.

a

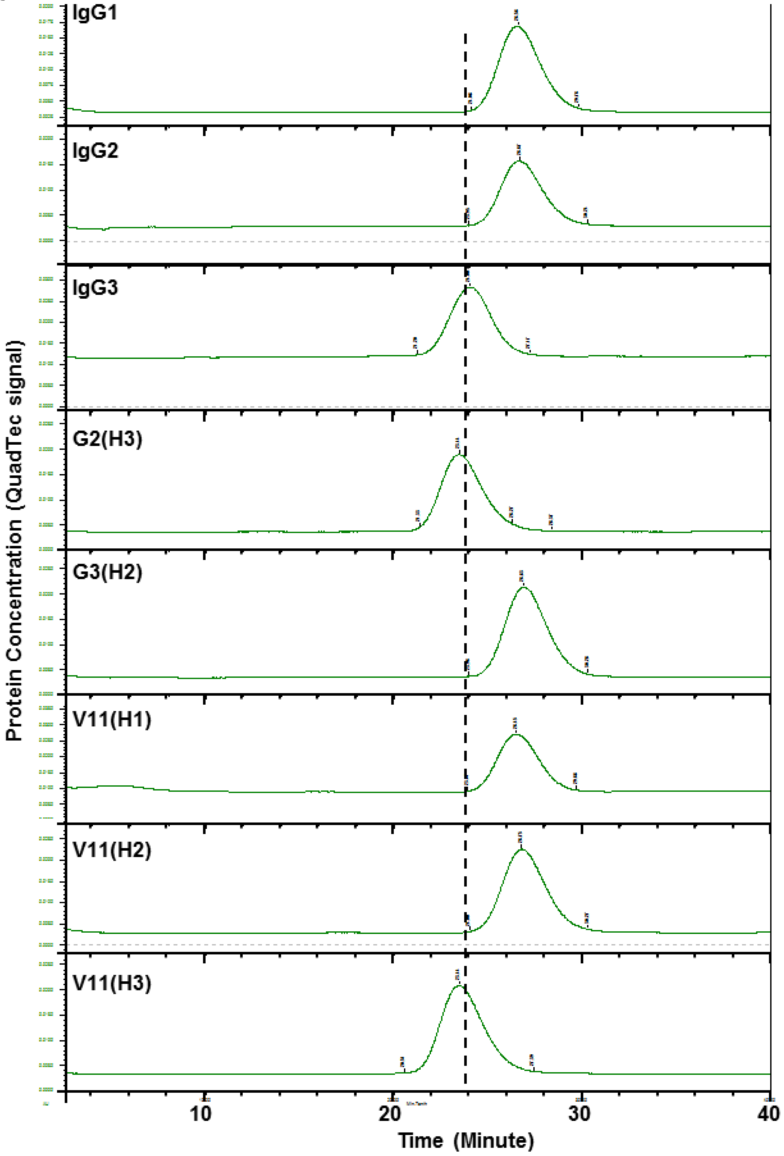
IgG1
IgG2
IgG3
G2(H3)
G3(H2)
V11(H1)
V11(H2)
V11(H3)
Protein Concentration (QuadTec signal)
10
20
30
40
Time (Minute)

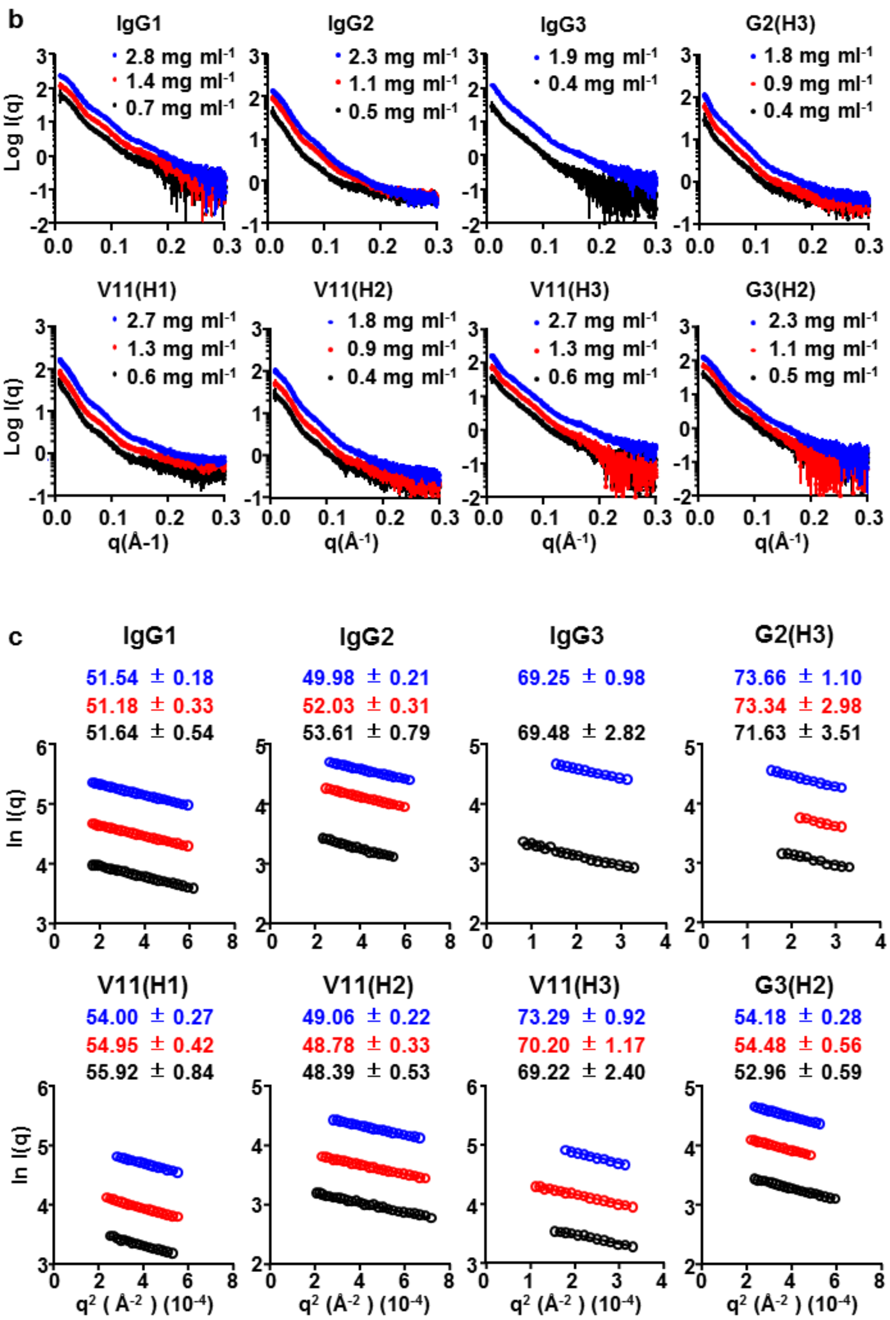
b
IgG1
2.8 mg ml-1
1.4 mg ml-1
0.7 mg ml-1
IgG2
2.3 mg ml-1
1.1 mg ml-1
0.5 mg ml-1
IgG3
1.9 mg ml-1
0.4 mg ml-1
G2(H3)
1.8 mg ml-1
0.9 mg ml-1
0.4 mg ml-1
V11(H1)
2.7 mg ml-1
1.3 mg ml-1
0.6 mg ml-1
V11(H2)
1.8 mg ml-1
0.9 mg ml-1
0.4 mg ml-1
V11(H3)
2.7 mg ml-1
1.3 mg ml-1
0.6 mg ml-1
G3(H2)
2.3 mg ml-1
1.1 mg ml-1
0.5 mg ml-1
Log I(q)
q(Å-1)
c
IgG1
51.54 ± 0.18
51.18 ± 0.33
51.64 ± 0.54
IgG2
49.98 ± 0.21
52.03 ± 0.31
53.61 ± 0.79
IgG3
69.25 ± 0.98
69.48 ± 2.82
G2(H3)
73.66 ± 1.10
73.34 ± 2.98
71.63 ± 3.51
V11(H1)
54.00 ± 0.27
54.95 ± 0.42
55.92 ± 0.84
V11(H2)
49.06 ± 0.22
48.78 ± 0.33
48.39 ± 0.53
V11(H3)
73.29 ± 0.92
70.20 ± 1.17
69.22 ± 2.40
G3(H2)
54.18 ± 0.28
54.48 ± 0.56
52.96 ± 0.59
ln I(q)
q2 (Å-2) (10-4)

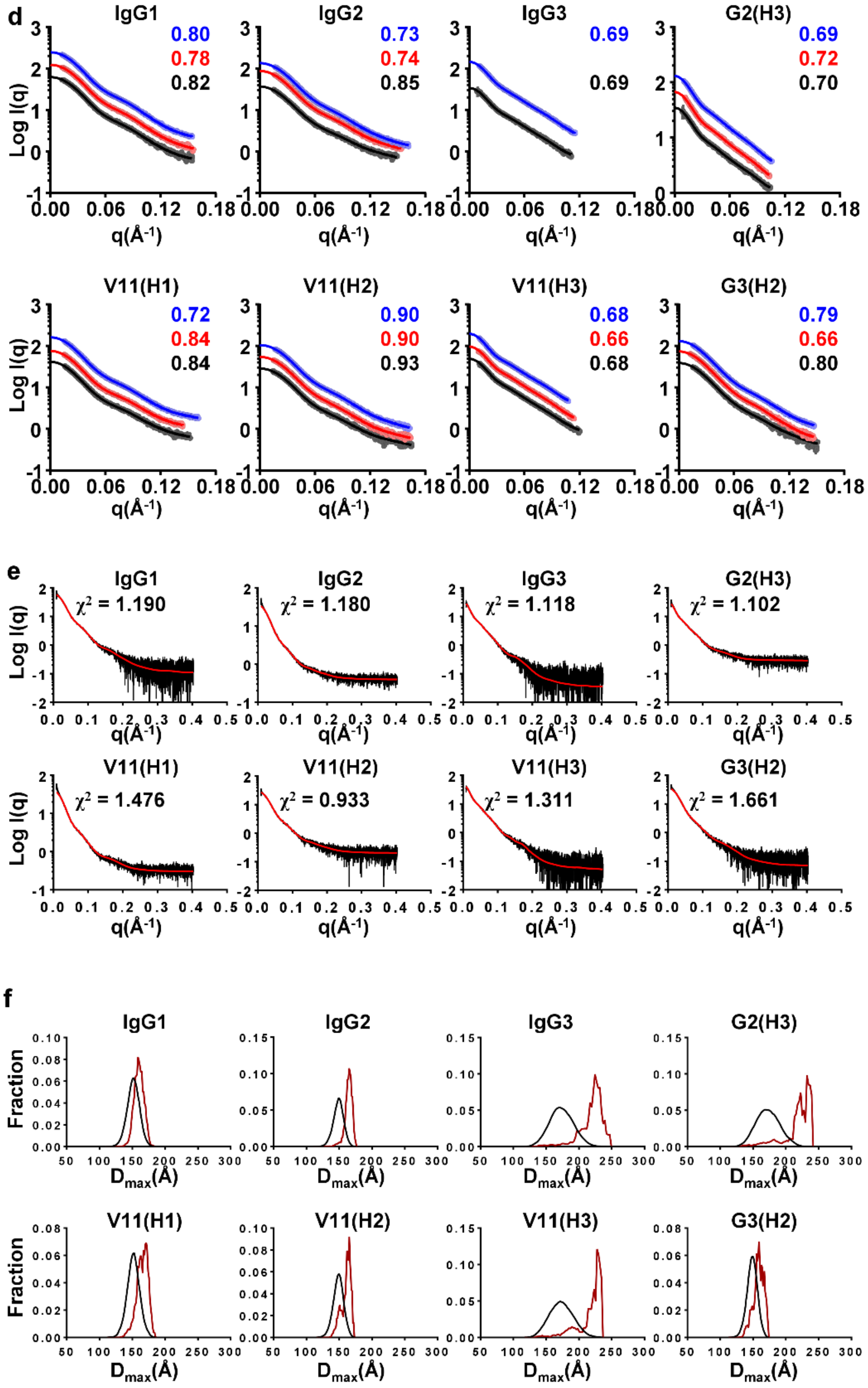
d
IgG1
0.80
0.78
0.82
IgG2
0.73
0.74
0.85
IgG3
0.69
0.69
G2(H3)
0.69
0.72
0.70
V11(H1)
0.72
0.84
0.84
V11(H2)
0.90
0.90
0.93
V11(H3)
0.68
0.66
0.68
G3(H2)
0.79
0.66
0.80
Log I(q)
q(Å-1)
e
IgG1
χ2 = 1.190
IgG2
χ2 = 1.180
IgG3
χ2 = 1.118
G2(H3)
χ2 = 1.102
V11(H1)
χ2 = 1.476
V11(H2)
χ2 = 0.933
V11(H3)
χ2 = 1.311
G3(H2)
χ2 = 1.661
Log I(q)
q(Å-1)
f
IgG1
IgG2
IgG3
G2(H3)
V11(H1)
V11(H2)
V11(H3)
G3(H2)
Fraction
Dmax(Å)

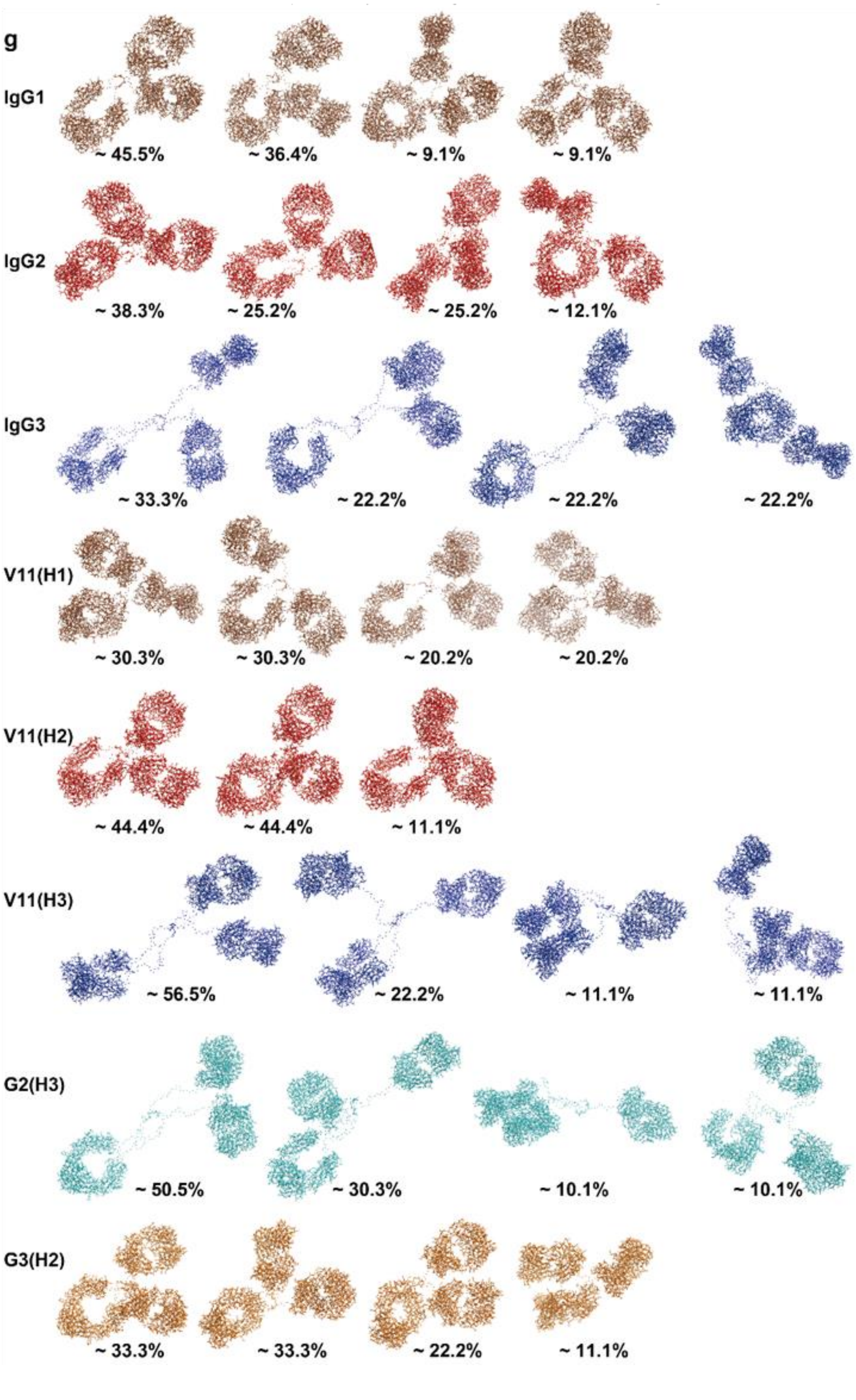
g
IgG1
~ 45.5%
~ 36.4%
~ 9.1%
~ 9.1%
IgG2
~ 38.3%
~ 25.2%
~ 25.2%
~ 12.1%
IgG3
~ 33.3%
~ 22.2%
~ 22.2%
~ 22.2%
V11(H1)
~ 30.3%
~ 30.3%
~ 20.2%
~ 20.2%
V11(H2)
~ 44.4%
~ 44.4%
~ 11.1%
V11(H3)
~ 56.5%
~ 22.2%
~ 11.1%
~ 11.1%
G2(H3)
~ 50.5%
~ 30.3%
~ 10.1%
~ 10.1%
G3(H2)
~ 33.3%
~ 33.3%
~ 22.2%
~ 11.1%

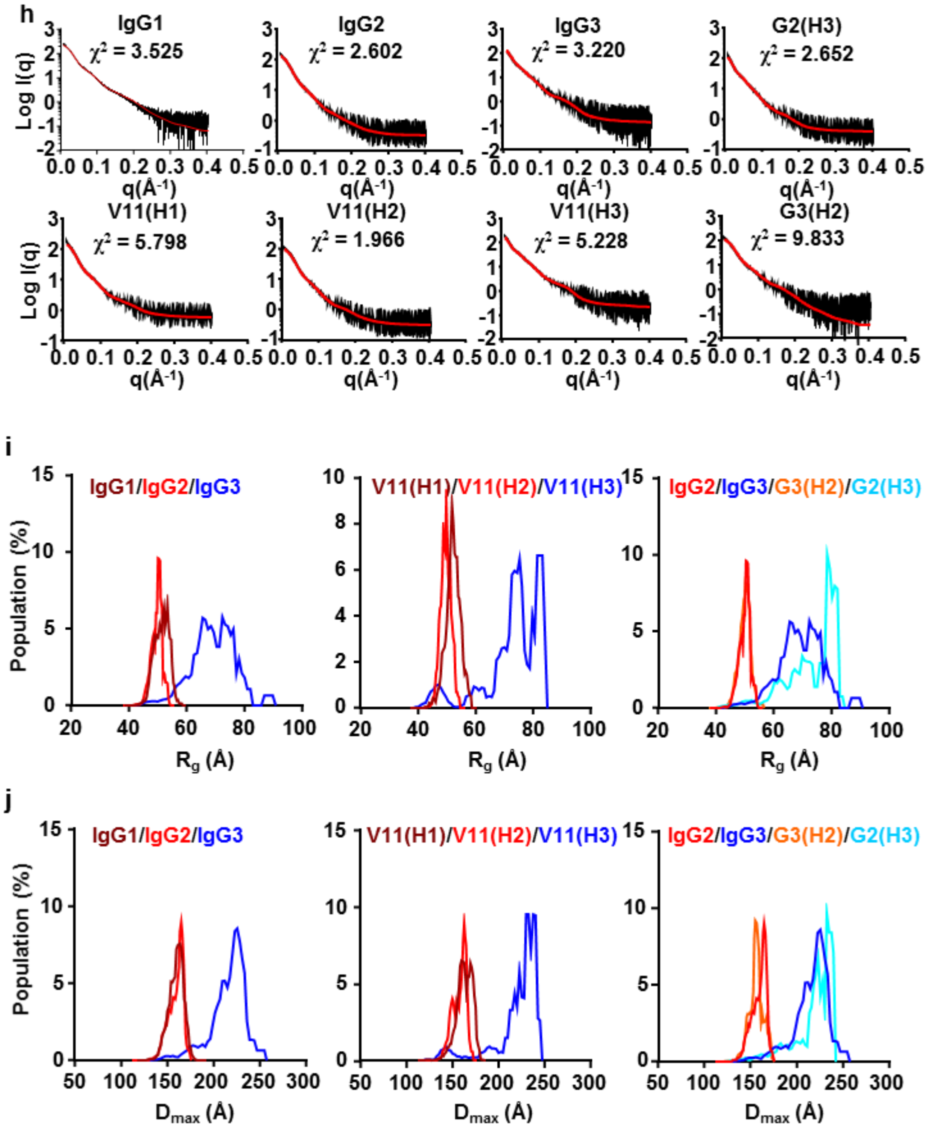
h
IgG1
χ² = 3.525
IgG2
χ² = 2.602
IgG3
χ² = 3.220
G2(H3)
χ² = 2.652
V11(H1)
χ² = 5.798
V11(H2)
χ² = 1.966
V11(H3)
χ² = 5.228
G3(H2)
χ² = 9.833
Log I(q)
q(Å⁻¹)
i
IgG1/IgG2/IgG3
V11(H1)/V11(H2)/V11(H3)
IgG2/IgG3/G3(H2)/G2(H3)
Population (%)
Rg (Å)
j
IgG1/IgG2/IgG3
V11(H1)/V11(H2)/V11(H3)
IgG2/IgG3/G3(H2)/G2(H3)
Population (%)
Dmax (Å)

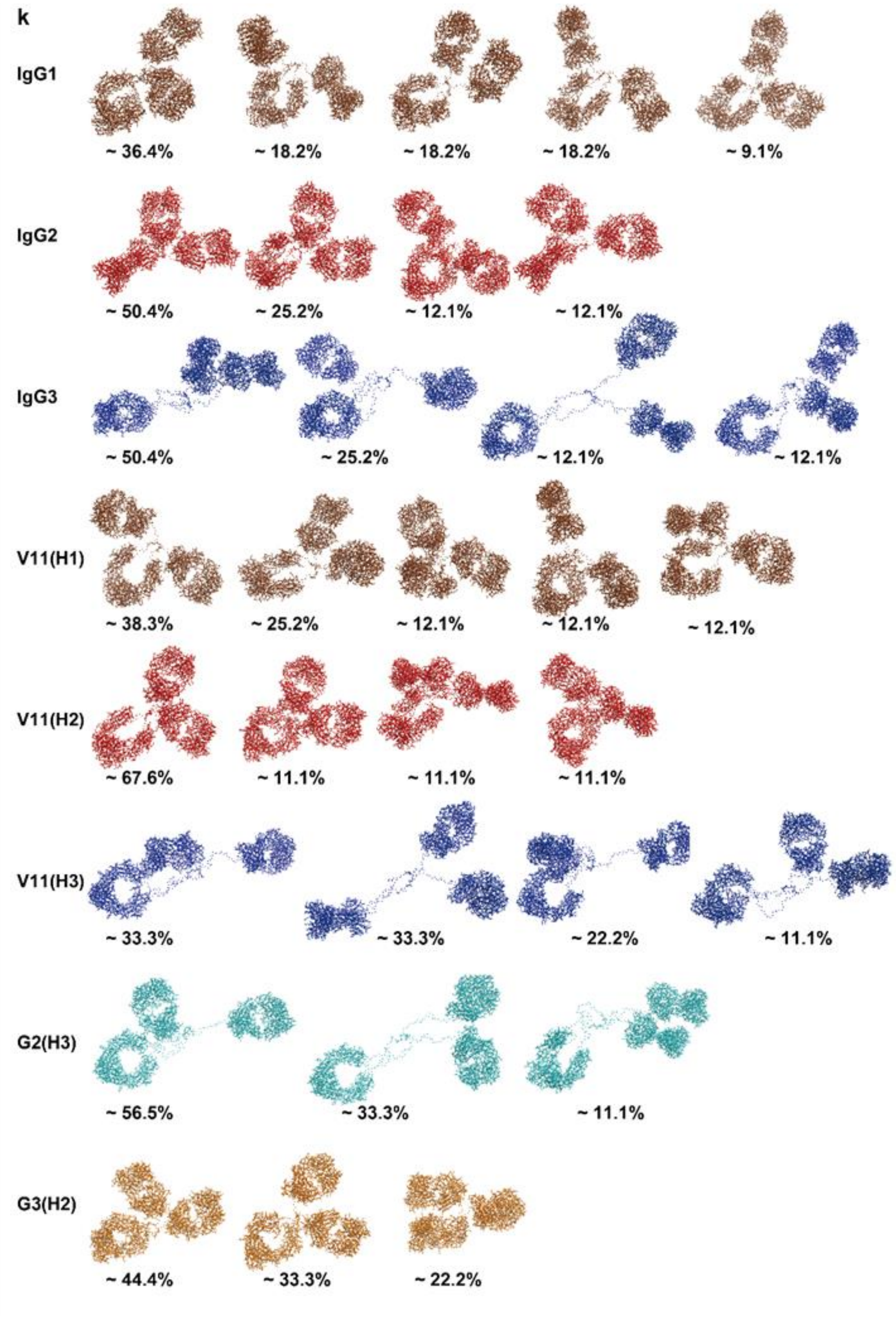

Supplementary Fig. 6. Small-angle X-ray scattering (SAXS) analysis of anti-CD40 antibodies. (**a**) Size-Exclusion Chromatography (SEC) profiles of anti-mouse CD40 antibodies of the indicated constant domains separated with a Superdex200 column at a flow rate of 0.5 ml minute^{-1}. **(b)** Scattering intensity plots (Log I(q) versus q) of serial-diluted anti-CD40 antibodies of indicated constant domains were collected at labelled concentrations. (**c**) Guinier plots (Ln I(q) vs

q^2) of antibodies in (**b**), with Rg values (Mean $\pm$ SD) annotated (colour-coded as in (**b**): blue, high concentration; red, middle concentration; black, low concentration). (**d**) Fitting of computed (solid lines) to experimental (dots) scattering intensity (Log (Iq)) plots of serially diluted antibody samples in (**b**) and (**c**) (colour-coded as in (**b**) and (**c**)) in distant distribution analysis (P(R)) by GNOM**.** Quality scores are labelled. **(e)** Fits between the calculated scattering curve from the best ensemble (red line, selected by EOM) and the experimental data (black line) (at the lowest tested concentrations). **(f)** D_{max} distributions of the ensemble pool (black) and the optimized ensembles (red) of indicated antibodies (at the lowest tested concentrations). **(g)** Models composing the best fitting ensemble generated by the EOM analysis of indicated antibodies (at the lowest tested concentrations), with the Fc domains pointing to the lower-left direction and the overall occurrence noted. **(h)** Fits between the calculated scattering curve from the best ensemble (red line, selected by EOM) and the experimental data (black line) (at the highest tested concentrations). (**i, j**) The distribution of R_g **(i)** and D_{max} **(j)** in the optimized ensembles generated in EOM analysis of the indicated antibodies (at the highest tested concentrations). **(k)** Models composing the best fitting ensemble generated by the EOM analysis of indicated antibodies (at the highest tested concentrations).

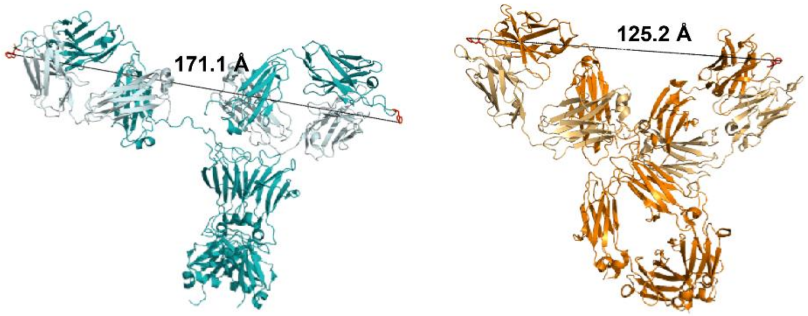

Supplementary Fig. 7. A distance over 12 nm between Ag binding sites in crystalized full human IgG antibodies. The overall structure of human IgG1 (PDB:1HZH, coloured teal) and IgG4 (PDB: 5DK3, coloured orange). The distances between two Fabs are measured from the apex of one heavy chain CDR3 site (W100 in 1HZH and F103 in 5D3K, coloured red) to the other in PyMOL (http://www.pymol.org/).

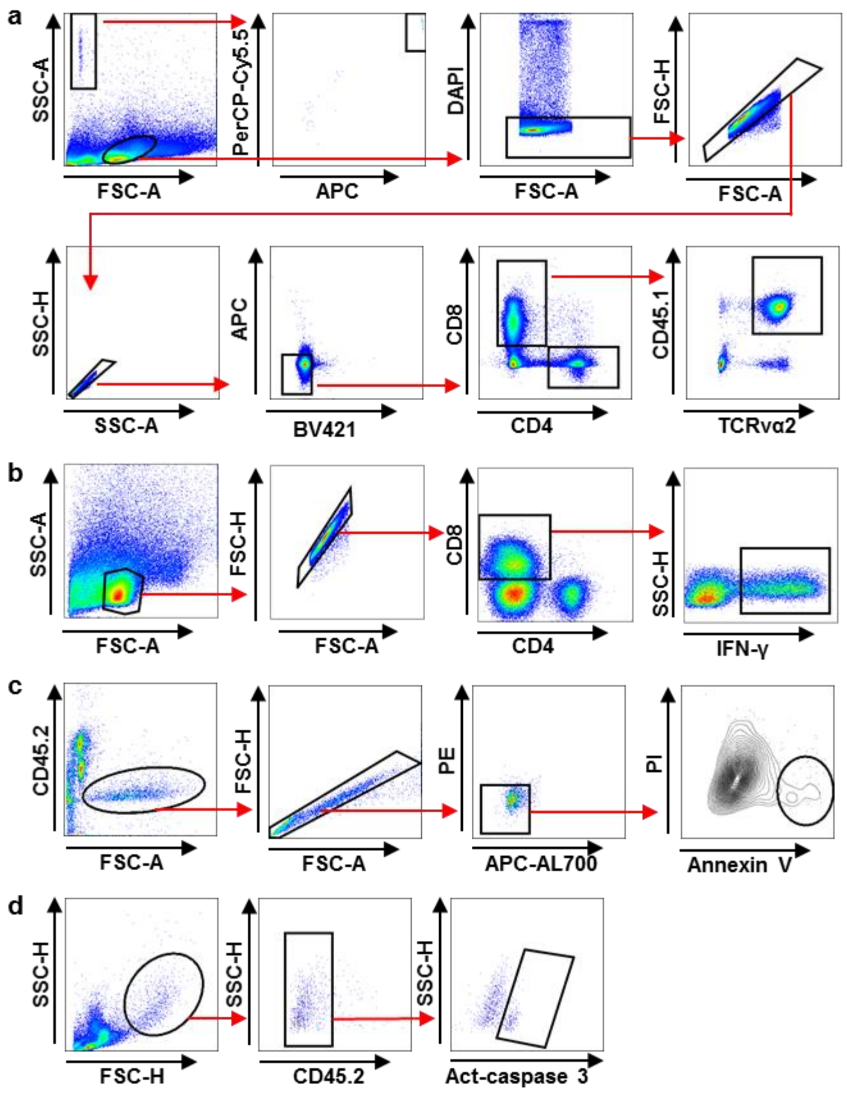

Supplementary Fig. 8. Gating strategy for Flow-cytometry analysis. (a) Gating strategy of OVA-specific CD8$^+$ T cells (CD8$^+$CD4$^-$CD45.1$^+$TCRVα2$^+$) on day 6 in mouse spleen cells (BV421 and APC are non-staining channels) used to quantify OVA-specific CD8+ T cells presented in Figs.1b-g, 2a-f, 6b-c, Supplementary Fig. 2b, Supplementary Fig. 2c, and Supplementary Fig. 4a. **(b)** Gating strategy of IFN-γ$^+$ CD8$^+$ T cells (CD8$^+$CD4$^-$IFN-γ$^+$) upon OVA peptide SIINFEKL stimulation in splenocytes presented in Supplementary Fig. 2a and Supplementary Fig. 4b. **(c)** Gating strategy for pro-apoptotic (CD45.2$^-$Annexin V$^+$PI$^-$) MC38 cells presented in Figs. 7a-b. (PE and APC-AL700 are non-staining channels). **(d)** Gating strategy for pro-apoptotic (CD45.2$^-$Actived caspase3$^+$) MC38 cells presented in Fig. 7c.

Supplementary Table 1. Binding epitopes of CD40 antibodies in relationship to CD40L binding sites.

Specificity	Clone	Binding epitope	References
Mouse CD40	1C10	Block CD40L binding	26
Human CD40	21.4.1	CRD1 of hCD40; do not block hCD40L binding	26, 27,
Human CD40	3.1.1	CRD2/3 of hCD40; block hCD40L binding	26, 27

CRD, cysteine-rich domain

Supplementary Table 2. Hinge and Fc sequences of various human IgG constant domains.

Constant domains	C-terminal of CH1	Upper hinge	Middle hinge	Hinge length	Fc
G1	NTKVDKRV	EPKSCDKTHT	CPPCP	15	G1
G2	NTKVDKTV	ERK	CCVECPPCP	12	G2
G2(GS)$_3$	NTKVDKTV	ERKGSGSGS	CCVECPPCP	18	G2
G3(H2)	NTKVDKTV	ERK	CCVECPPCP	12	G3
G3	NTKVDKRV	ELKTPLGDTTHT	CPRCP(EPKSCDTPPPCPRCP)$_3$	62	G3
G2(H3)	NTKVDKRV	ELKTPLGDTTHT	CPRCP(EPKSCDTPPPCPRCP)$_3$	62	G2
G4	NTKVDKRV	ESKYGPP	CPSCP	12	G4
V11(H1)	NTKVDKRV	EPKSCDKTHT	CPPCP	15	V11
V11(H2)	NTKVDKTV	ERK	CCVECPPCP	12	V11
V11(H3)	NTKVDKRV	ELKTPLGDTTHT	CPRCP(EPKSCDTPPPCPRCP)$_3$	62	V11

Supplementary Table 3. The condition of SAXS Data-collection and analysis

(a) SAXS data-collection parameters

Instrument/data processing	The beam line BL19U2 of National Center for Protein Science Shanghai (NCPSS) at Shanghai Synchrotron Radiation Facility (SSRF)
Wavelength (Å)	1.033
Beam size (mm)	0.40 x 0.15
q measurement range ($Å^{-1}$)	0.0086-0.40307
Normalization	To transmitted intensity by beam-stop counter
Monitoring for radiation damage	To reduce the radiation damage, a flow cell made of a cylindrical quartz capillary with a diameter of 1.5 mm and a wall of 10 µm was used.
Exposure time	Continuous 1 second
Sample configuration	Sixty µl of each sample was continuously passed through a capillary tube exposed Measurements were carried out at 3 different concentrations in all cases (0.4-2.8mg/ml)
Sample temperature (℃)	25

(b) The software employed for SAXS data reduction, analysis, interpretation, and modelling

SAXS data reduction	BioXTAS RAW (version 1.2.1)
Basic analyses: Rg, I_0, Guinier	The AUTORG program (the “Radius of Gyration” function of the program PRIMUSQT from the ATSAS program suite (Version 2.8.4))
Basic analyses: P(r), V_P	The GNOM program (the "Distance Distribution” function of the program PRIMUSQT from the ATSAS program suite (Version 2.8.4))
Ensemble modelling	The Ensemble Optimization Method (EOM 2.1)
Homology modelling	The SWISS-MODEL Workspace
PDB file processing	The PyMOL Molecular Graphic System (version 1.7.2.1, Schrodinger LLC.)

Supplementary Table 4. EOM results of high-concentration SAXS samples (default parameters, 10,000 models in the initial ensemble, native-like models, constant subtraction allowed).

	G1	G2	G3	V11(H1)	V11(H2)	V11(H3)	G2(H3)	G3(H2)
χ^2	3.525	2.602	3.220	5.798	1.966	5.228	2.652	9.833
No. of curves.	11	8	8	8	9	9	9	9
R_{flex} (random) (%)	~ 81.05 (~ 84.33)	~ 77.15 (~ 84.16)	~ 85.21 (~ 80.87)	~ 79.06 (~ 82.15)	~ 76.63 (~ 83.43)	~ 82.99 (~ 83.55)	~ 83.45 (~ 83.33)	~ 78.43 (~ 83.40)
R_{δ}	0.89	0.79	1.33	0.90	0.76	1.68	1.50	0.84
