# Supplementary material for: Human immunoglobulin G hinge regulates agonistic anti-CD40 immunostimulatory and antitumour activities through biophysical flexibility
Source: Nat Commun. 2019 Sep 27;10:4206. doi: 10.1038/s41467-019-12097-6 (PMC6765011; doi:10.1038/s41467-019-12097-6)
Supplement: Supplementary file 3 — Reporting Summary [file 41467_2019_12097_MOESM3_ESM.pdf]

nature research

Corresponding author(s): Fubin Li (fubin.li@sjtu.edu.cn)

Reporting Summary

Nature Research wishes to improve the reproducibility of the work that we publish. This form provides structure for consistency and transparency in reporting. For further information on Nature Research policies, see Authors & Referees and the Editorial Policy Checklist.

Statistical parameters

When statistical analyses are reported, confirm that the following items are present in the relevant location (e.g. figure legend, table legend, main text, or Methods section).

n/a	Confirmed	
☐	☒	The exact sample size (*n*) for each experimental group/condition, given as a discrete number and unit of measurement
☐	☒	An indication of whether measurements were taken from distinct samples or whether the same sample was measured repeatedly
☐	☒	The statistical test(s) used AND whether they are one- or two-sided<br>*Only common tests should be described solely by name; describe more complex techniques in the Methods section.*
☒	☐	A description of all covariates tested
☒	☐	A description of any assumptions or corrections, such as tests of normality and adjustment for multiple comparisons
☒	☐	A full description of the statistics including central tendency (e.g. means) or other basic estimates (e.g. regression coefficient) AND variation (e.g. standard deviation) or associated estimates of uncertainty (e.g. confidence intervals)
☒	☐	For null hypothesis testing, the test statistic (e.g. *F*, *t*, *r*) with confidence intervals, effect sizes, degrees of freedom and *P* value noted<br>*Give P values as exact values whenever suitable.*
☒	☐	For Bayesian analysis, information on the choice of priors and Markov chain Monte Carlo settings
☒	☐	For hierarchical and complex designs, identification of the appropriate level for tests and full reporting of outcomes
☒	☐	Estimates of effect sizes (e.g. Cohen's *d*, Pearson's *r*), indicating how they were calculated
☐	☒	Clearly defined error bars<br>*State explicitly what error bars represent (e.g. SD, SE, CI)*

Our web collection on statistics for biologists may be useful.

Software and code

Policy information about availability of computer code

Data collection	BD FACSDiva Software
Data analysis	Flowjo X (Version 10.0.7r2); Prism 6 for Windows (version 6.01); BioXTAS RAW (Version 1.2.1); the ATSAS program suite (Version 2.8.4); The PyMOL Molecular Graphic System (version 1.7.2.1, Schrodinger LLC.); The SWISS-MODEL Workspace

For manuscripts utilizing custom algorithms or software that are central to the research but not yet described in published literature, software must be made available to editors/reviewers upon request. We strongly encourage code deposition in a community repository (e.g. GitHub). See the Nature Research guidelines for submitting code & software for further information.

Data

Policy information about availability of data

All manuscripts must include a data availability statement. This statement should provide the following information, where applicable:

- Accession codes, unique identifiers, or web links for publicly available datasets
- A list of figures that have associated raw data
- A description of any restrictions on data availability

All data generated or analyzed during this study are either included in this manuscript (Figures and supplementary information) or available upon reasonable request. A section titled “Data Availability” is included in the manuscript after the Methods section and before the References.

April 2018

Field-specific reporting

Please select the best fit for your research. If you are not sure, read the appropriate sections before making your selection.

☒ Life sciences ☐ Behavioural & social sciences ☐ Ecological, evolutionary & environmental sciences

For a reference copy of the document with all sections, see nature.com/authors/policies/ReportingSummary-flat.pdf

Life sciences study design

All studies must disclose on these points even when the disclosure is negative.

Sample size	No statistical method was used to predetermine sample sizes. For most in vivo studies, sample sizes that are more than 3 were chosen to overcome individual variation as in common practice. These sample sizes are sufficient because significant differences were consistently observed.
Data exclusions	No data were excluded.
Replication	Reproducibility of the experimental findings were verified by repeated experiments (all major conclusions were supported by at least two independent experiments). All attempts at replication were successful.
Randomization	Mice were randomly allocated into different groups.
Blinding	Investigators were blinded to group allocation during data analysis.

Reporting for specific materials, systems and methods

Materials & experimental systems

n/a	Involved in the study	
☒	☐	Unique biological materials
☐	☒	Antibodies
☐	☒	Eukaryotic cell lines
☒	☐	Palaeontology
☐	☒	Animals and other organisms
☒	☐	Human research participants

Methods

n/a	Involved in the study	
☒	☐	ChIP-seq
☐	☒	Flow cytometry
☒	☐	MRI-based neuroimaging

Antibodies

Antibodies used	All antibodies used in the study were described in the "Methods" section of the Supplementary Materials.
Validation	All antibodies used (commercially obtained or made in house) were validated either by manufacturers, others in cited references, or by the presented data in the manuscript.

Eukaryotic cell lines

Policy information about cell lines

Cell line source(s)	MC38 and MO4 cells are provided by Dr. Jeffrey Ravetch at the Rockefeller University
Authentication	None of the cell lines used were authenticated.
Mycoplasma contamination	None of the cell lines were tested for mycoplasma contamination.
Commonly misidentified lines (See ICLAC register)	N/A

Animals and other organisms

Policy information about studies involving animals; ARRIVE guidelines recommended for reporting animal research

Laboratory animals	All mice used in the study were described in the "Methods" section of the Supplementary Materials
Wild animals	N/A
Field-collected samples	N/A

Flow Cytometry

Plots

Confirm that:

☒ The axis labels state the marker and fluorochrome used (e.g. CD4-FITC).

☒ The axis scales are clearly visible. Include numbers along axes only for bottom left plot of group (a 'group' is an analysis of identical markers).

☒ All plots are contour plots with outliers or pseudocolor plots.

☒ A numerical value for number of cells or percentage (with statistics) is provided.

Methodology

Sample preparation	Method to perform flow cytometry is described in the "Flow cytometry" part of the "Methods" section.
Instrument	BD LSRFortessa™ X-20 analyzer (BD Biosciences), BD FACSCanto II (BD Biosciences), BD FACSCalibur (BD Biosciences).
Software	BD FACSDiva Software, Flowjo X (Version 10.0.7r2).
Cell population abundance	N/A
Gating strategy	Information for gating strategy is described in Supplementary Fig. 8, and the relevant "Results" and "Methods" section.

☒ Tick this box to confirm that a figure exemplifying the gating strategy is provided in the Supplementary Information.
